# Supplementary figures and images for: Casein Kinases 2-dependent phosphorylation of the placental ligand VAR2CSA regulates Plasmodium falciparum-infected erythrocytes cytoadhesion
Source: PLoS Pathog. 2025 Jan 13;21(1):e1012861. doi: 10.1371/journal.ppat.1012861 (PMC11761665; doi:10.1371/journal.ppat.1012861)

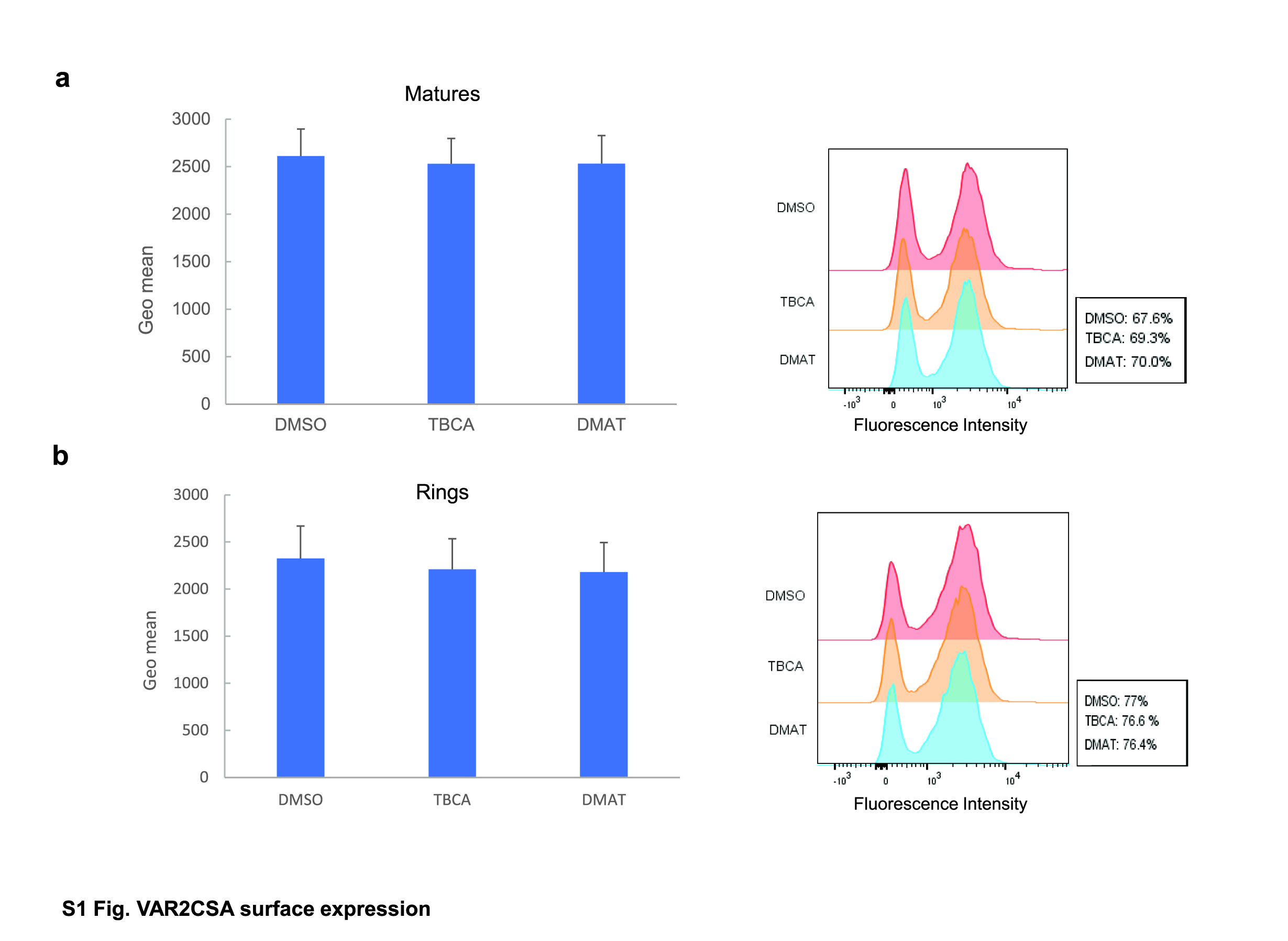

Supplement: S1 Fig — (a) VAR2CSA Surface expression of trophozoite IEs after one treatment with CK2 inhibitors or DMSO, monitored by flow cytometry with a specific anti-VAR2CSA antibody. (b) VAR2CSA surface expression of ring stage IEs after 16-hours treatment with CK2α inhibitors or DMSO monitored by flow cytometry with a specific anti-VAR2CSA antibody. Geometric means of fluorescence intensities of three independent experiments are represented with standard deviations. (TIF) [file ppat.1012861.s001.tif]

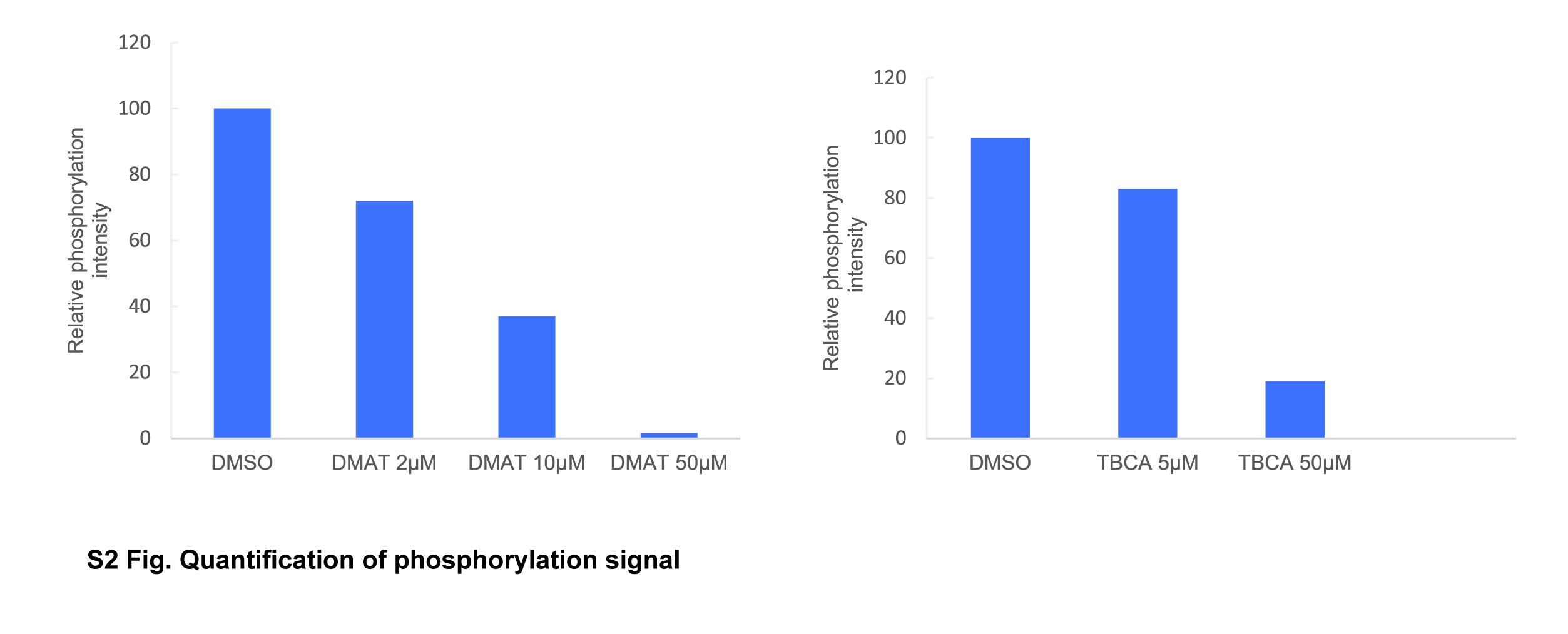

Supplement: S2 Fig — In vitro radioactive [γ—32P] ATP phosphorylation assays of recombinant His tagged VAR2CSA DBL1-6 protein (1μg in all assays) were performed in the presence of total IEs lysates and increasing concentrations of DMAT or TBCA. The phosphorylation signal for each condition was quantified by Image Lab Software and adjusted to reflect a percentage compared to signal obtained in DMSO control condition (100%). (TIF) [file ppat.1012861.s002.tif]

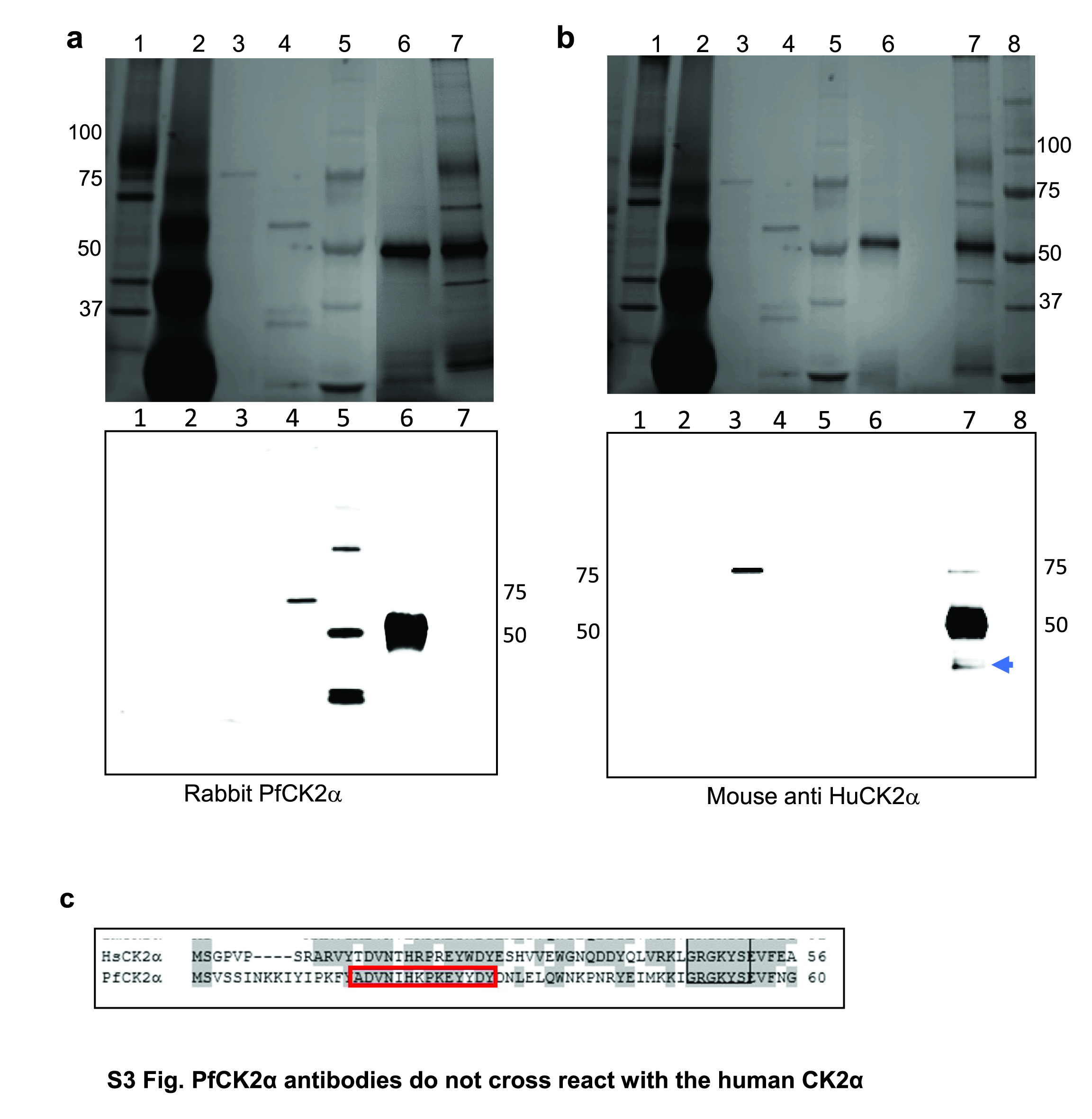

Supplement: S3 Fig — A rabbit polyclonal anti-PfCK2α and a mouse anti-HuCK2α were used in immunoprecipitation experiments performed on total uninfected RBC extracts. A fraction of the immunoprecipitated material was loaded on a stain free gel prior western blot. Upper panels are the stain free gels and lower panels are the western blots. Recombinant MBP-HuCK2α and GST-PfCK2α were loaded as controls. (a) Anti-PfCK2α western blot. Lane 1: uRBC membrane fractions; lane 2: total uRBC lysates. lane 3: MBP HuCK2α; lane 4: GST PfCK2α; lane 6: Immunoprecipitation with a rabbit anti PfCK2α; lane 7: immunoprecipitation with a mouse anti-Human CK2α. (b) Anti-HuCK2α western blot. Lane 1: uRBC membrane fractions; lane 2: total uRBC lysates. lane 3: MBP HuCK2α; lane 4: GST PfCK2α; lane 5: MW; lane 6: Immunoprecipitation with a rabbit anti PfCK2α; lane 7: immunoprecipitation with a mouse anti-Human CK2α; lane 8: MW. (c) Comparison of the N terminal of Pf and Hu CK2α protein sequences. The designed PfCK2α peptide used for rabbit immunization is boxed in red. (TIF) [file ppat.1012861.s003.tif]

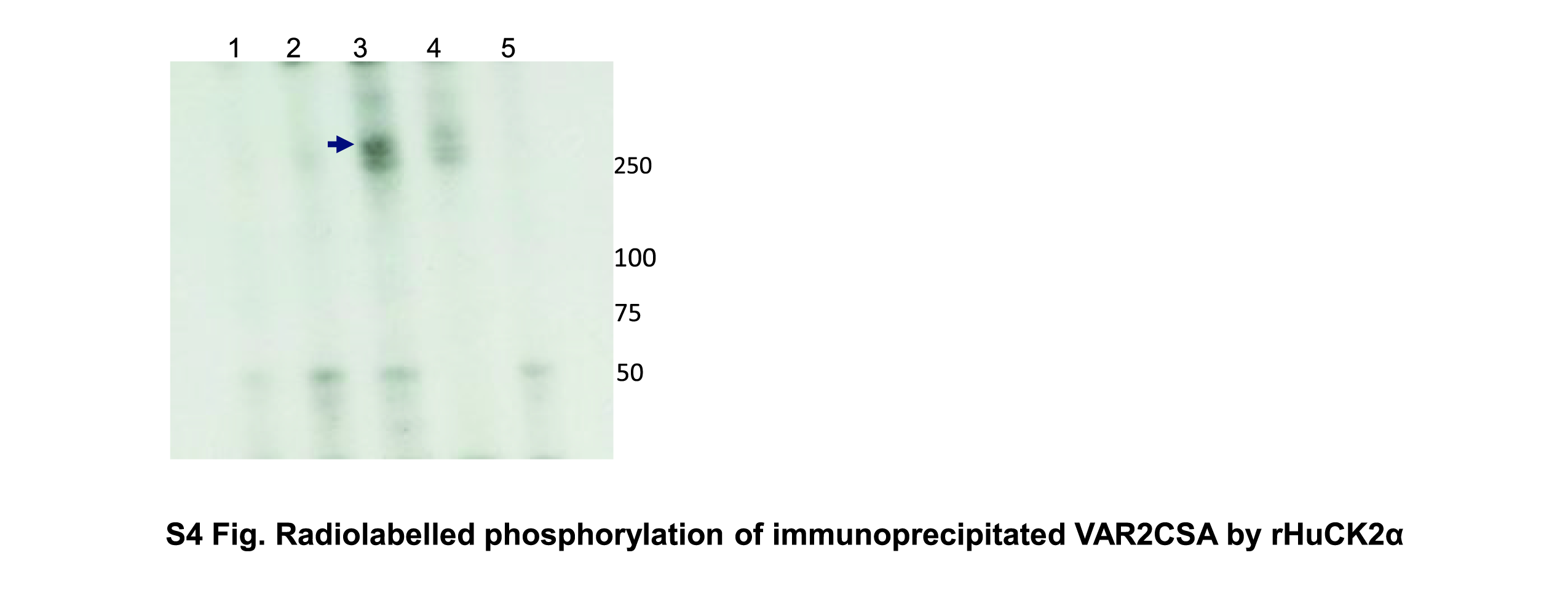

Supplement: S4 Fig — Immunoprecipitation of VAR2CSA from membrane fractions of uRBC and various selected VAR2CSA strains was followed by a radiolabelled phosphorylation assay with rHuCK2α (lane 1 to 4). The arrow indicates the immunoprecipitated VAR2CSA from the membrane IEs lysates. Lane 1: immunoprecipitation from uRBC membrane lysates; lane 2: immunoprecipitation from membrane lysates of 7G8CSA IEs; lane3: immunoprecipitation from membrane lysates of NF54CSA IEs; lane 4: immunoprecipitation from membrane lysates of FCR3CSA IEs; lane 5: immunoprecipitation from membrane lysates of NF54CSA IEs without rHuCK2α. (TIF) [file ppat.1012861.s004.tif]

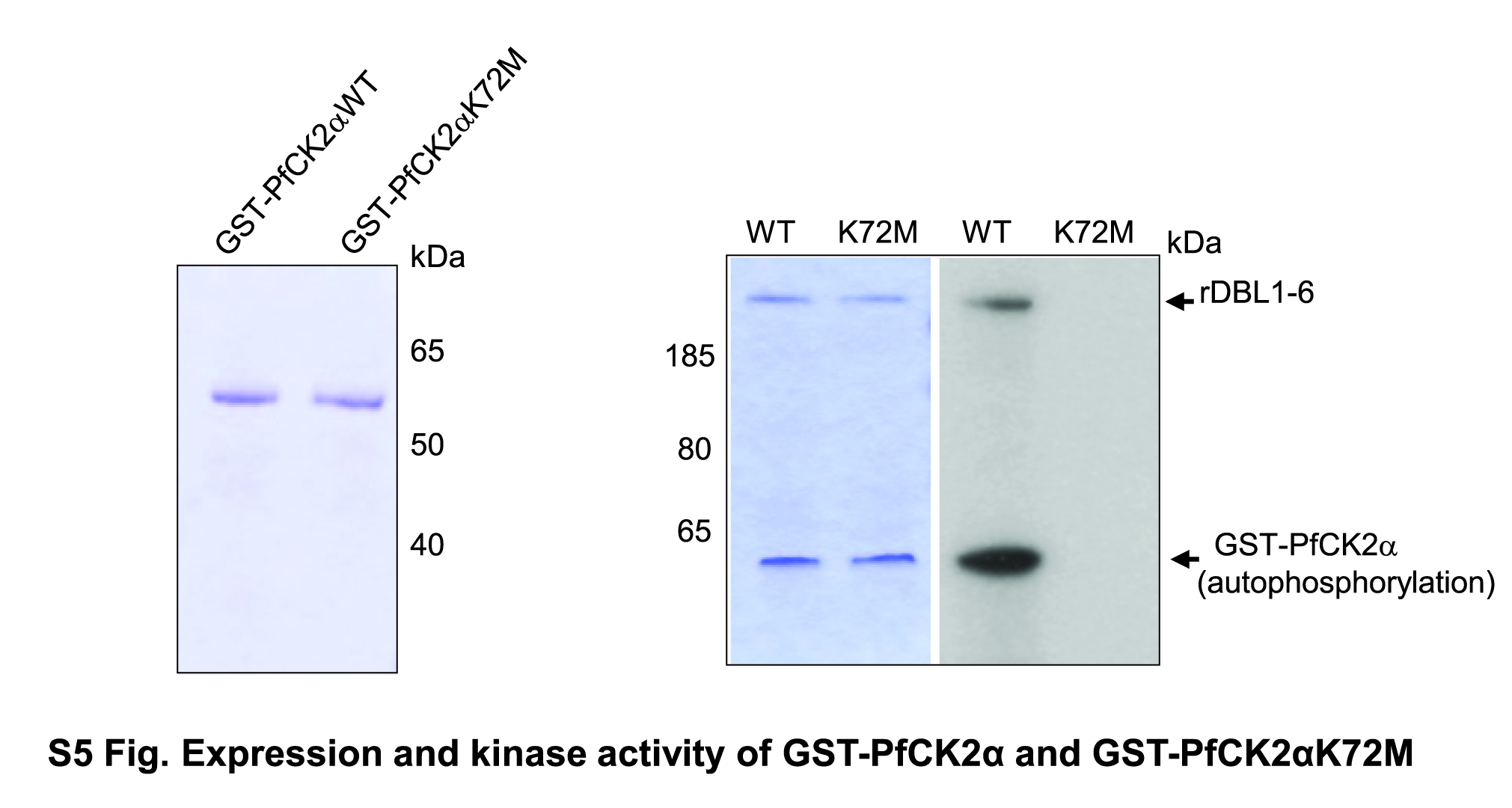

Supplement: S5 Fig — GST-PfCK2α and GST-PFCK2α K72M were expressed in E. Coli Rosetta and purified as described in 27. GST-PfCK2α kinase activity towards rDBL1-6. Autoradiograms (right) and Coomassie blue-stained gels (left) of kinase assays performed with GST-PfCK2α or catalytically inactive GST-K72MPfCK2α. The recombinant kinase and rDBL1-6 substrate are indicated with an arrow. Autophosphorylation of the wild-type kinase is shown. (TIF) [file ppat.1012861.s005.tif]

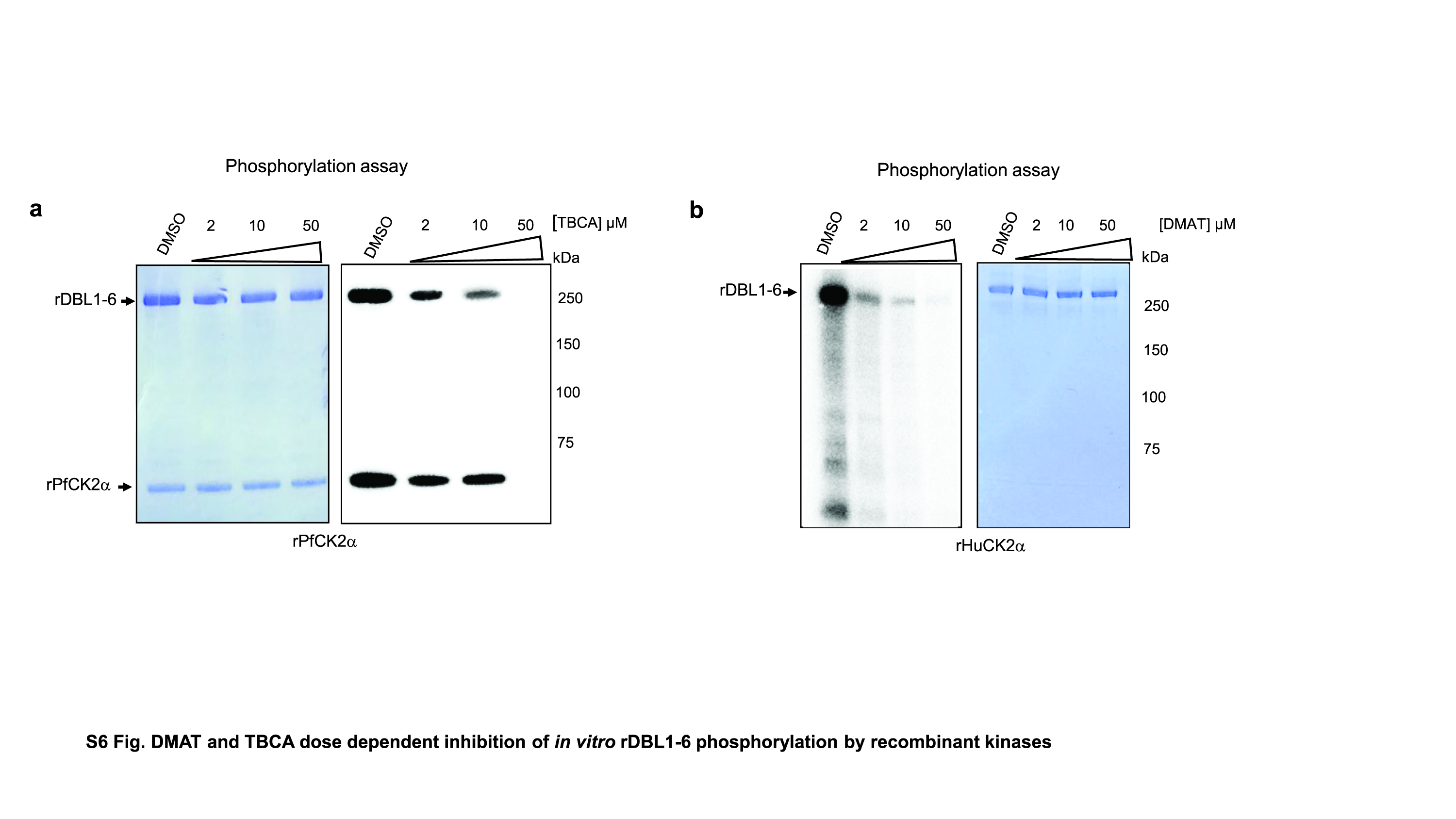

Supplement: S6 Fig — Recombinant DBL1-6 was used in in vitro phosphorylation assays in the presence of [γ—32P] ATP with recombinant Plasmodium CK2α or Human CK2α and with increasing concentrations of DMAT and TBCA. (a) (PfCK2α +TBCA): lane1: rDBL1-6 + DMSO; lane2: rDBL1-6 + TBCA 2μM; lane 3: rDBL1-6 + TBCA 10μM; lane 4: rDBL1-6 + TBCA 50μM; (b) (HuCK2α + DMAT): lane1: rDBL1-6 + DMSO; lane2: rDBL1-6 + DMAT 2μM; lane 3: rDBL1-6 + DMAT 10μM; lane 4: rDBL1-6 + DMAT 50μM. (TIF) [file ppat.1012861.s006.tif]

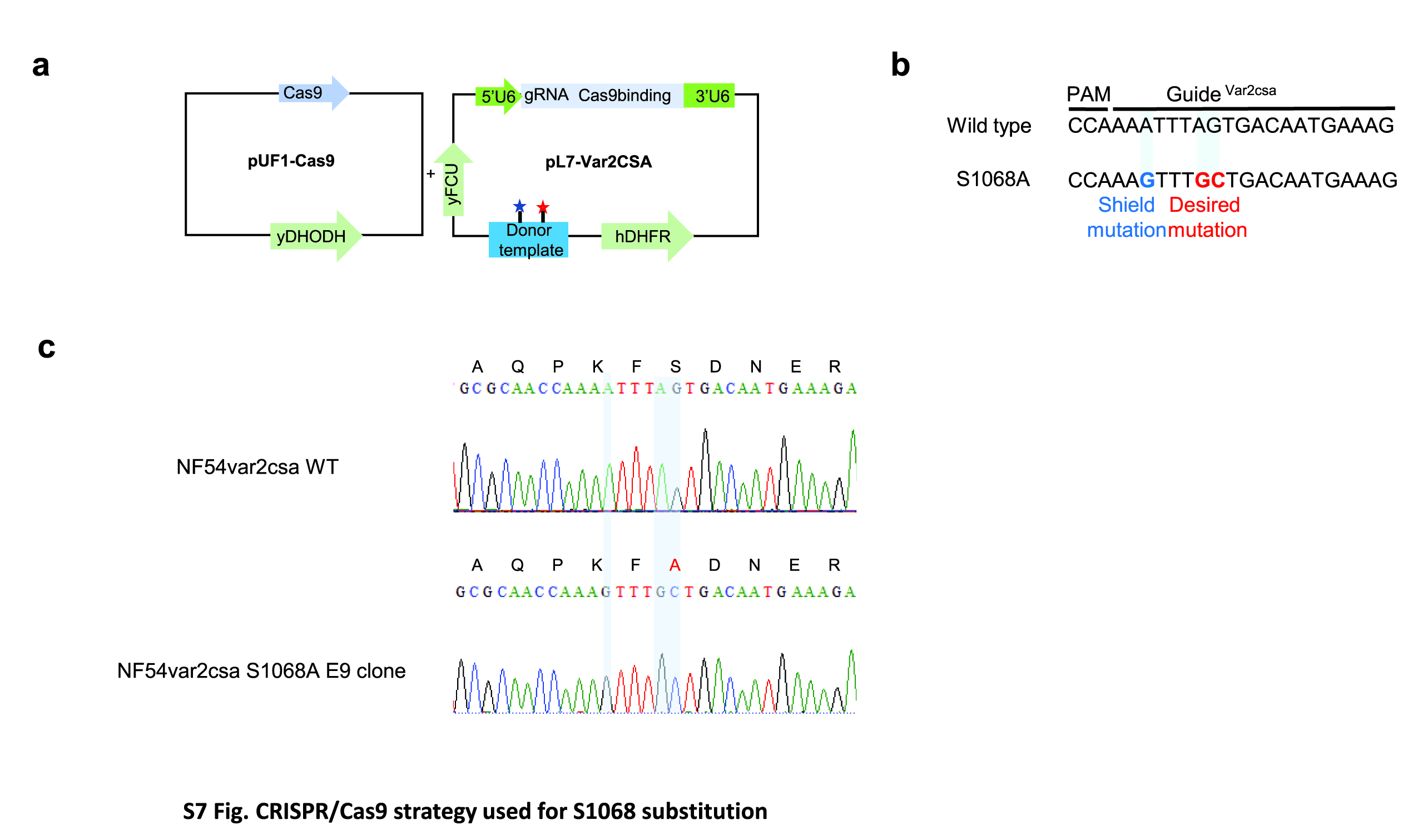

Supplement: S7 Fig — Nucleotide editing using sgRNA: Cas9 in P. falciparum. (a) Diagram illustrating the strategy used for nucleotide replacement. The Cas9 protein is expressed in the pUF1-Cas9 episome continuously maintained using the ydhodh drug-selectable marker. PL7- var2csa episome is maintained using the hDHFR selection and carries both the sgRNA var2csa and the donor DNA (blue box). The donor DNA carries the designed desired mutation (red star) and the shield mutation (blue star). (b) sgRNA var2csa targeted sequences recognized by Cas9. The 20 nucleotides guide and PAM sequences are indicated. (c) Chromatograms showing sequence analyses of var2csa locus in NF54CSA wild-type and in transgenic NF54 S1068A E9 clone. Nucleotide substitutions and amino acids changes in var2csa locus are highlighted. (TIF) [file ppat.1012861.s007.tif]

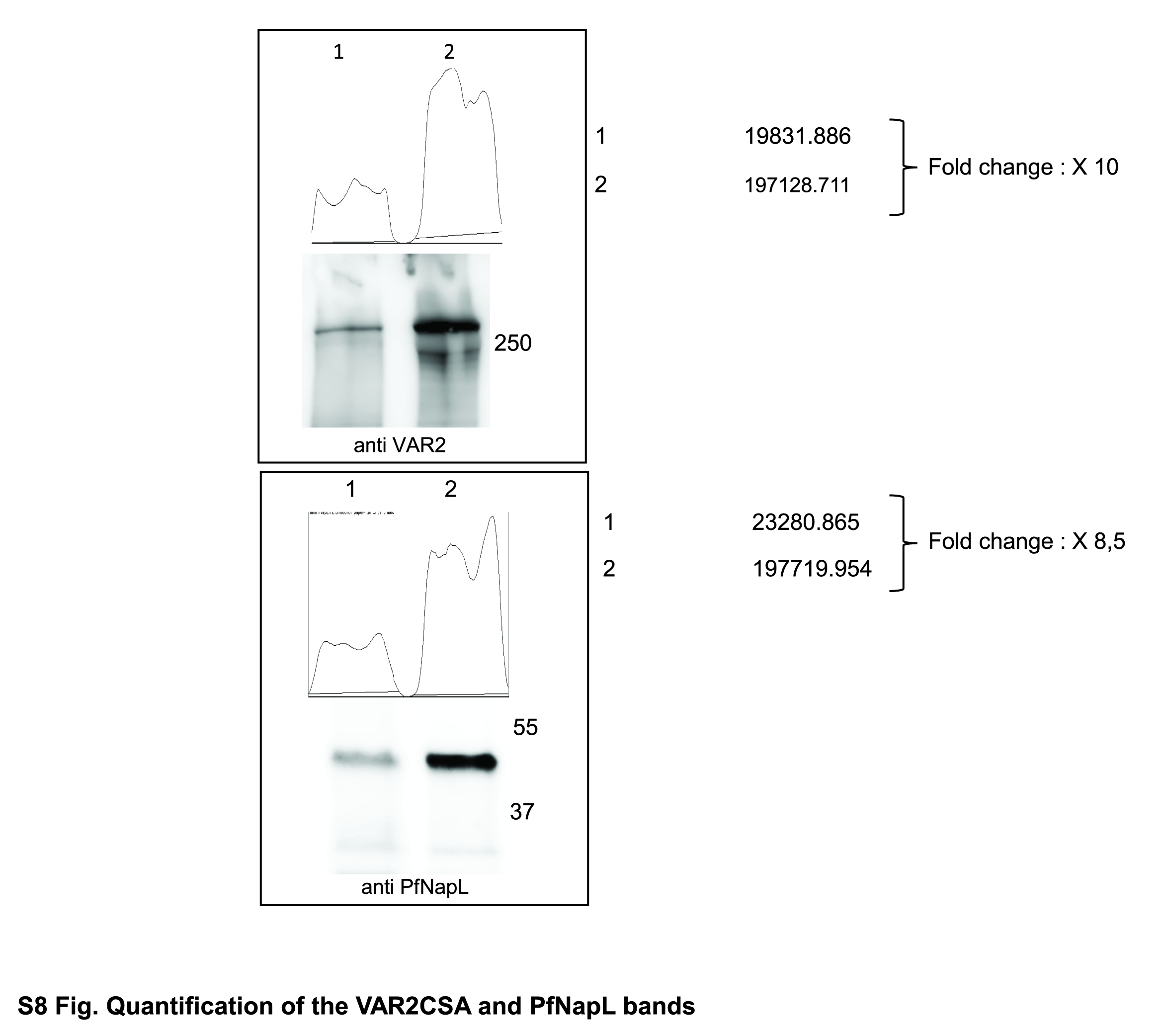

Supplement: S8 Fig — Quantification of the intensity of the bands of anti-VAR2CSA and anti-PfNapL western blots was performed by ImageJ software. (TIF) [file ppat.1012861.s008.tif]

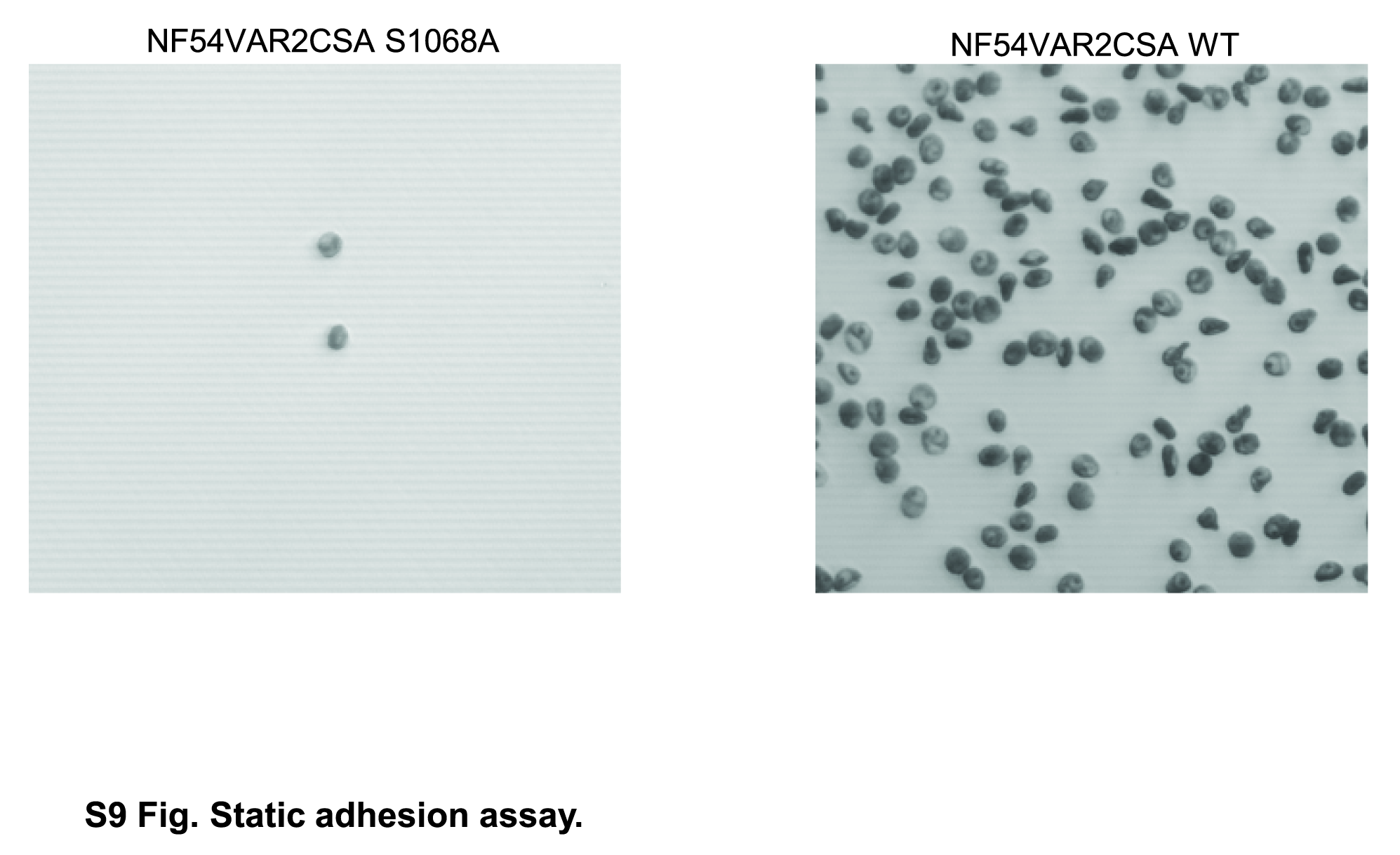

Supplement: S9 Fig — Image of a field showing bound NF54CSA parental and transgenic NF54 S1068A E9 clone IEs on CSA coated on plastic. (TIF) [file ppat.1012861.s009.tif]

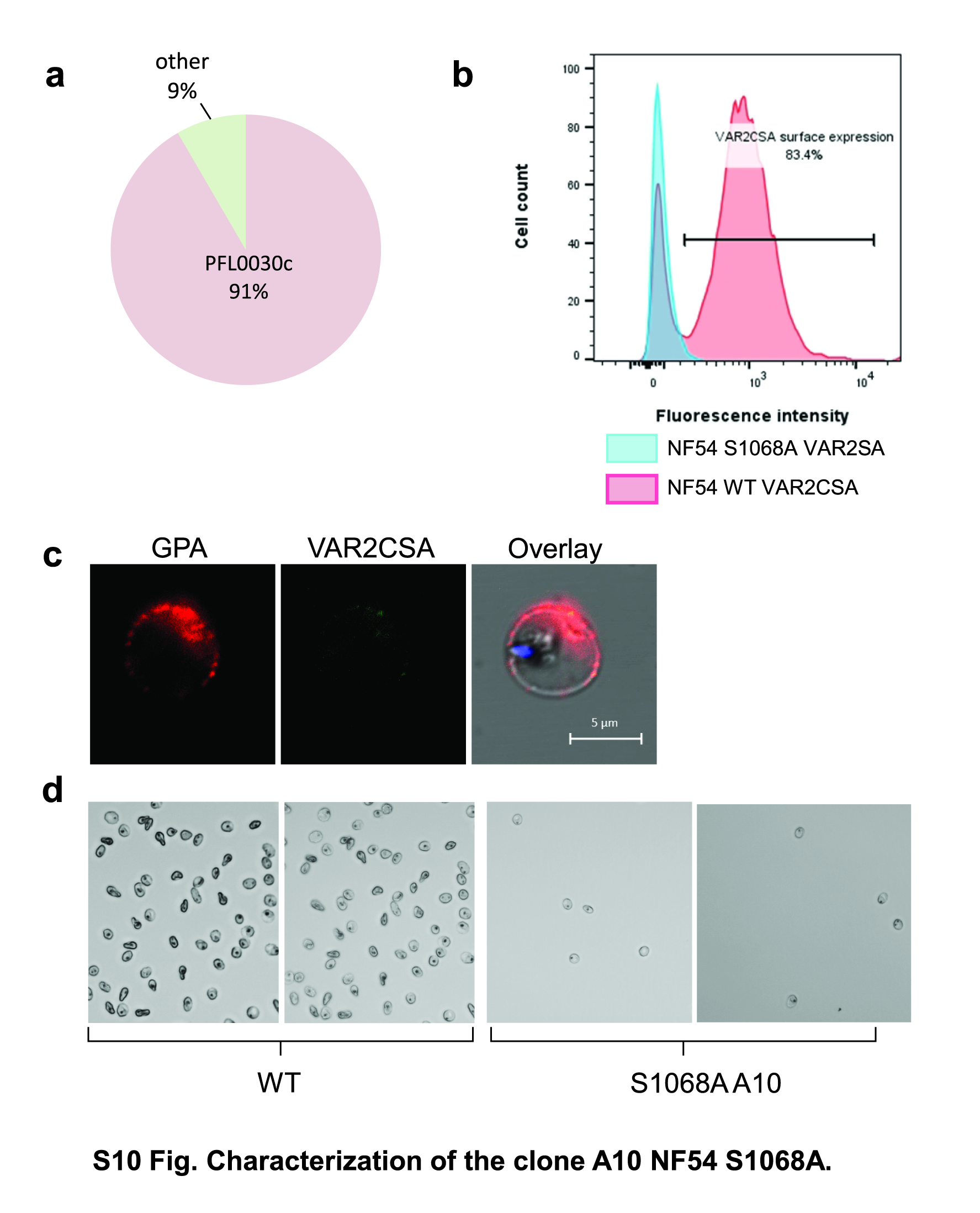

Supplement: S10 Fig — (a) Var transcriptional profile of S1068A clone A10 shown by qPCR. Transcriptional levels of each var genes were normalized with the housekeeping gene, seryl-tRNA transferase. (b). Flow cytometry analysis of wild-type and S1068A clone A10 NF54 IEs. IEs were labelled with rabbit anti-VAR2CSA antibodies. Geometric means of fluorescence intensities and percentage of IEs expressing VAR2CSA are indicated. (c) VAR2CSA immunofluorescence assays (IFA). IFA staining was performed on live cells on S1068A clone A10 NF54 IEs with rabbit anti-VAR2CSA and anti-GPA. (d) Static CSA cytoadhesion assay of wild-type and S1068A clone A10 NF54 IEs. (TIF) [file ppat.1012861.s010.tif]

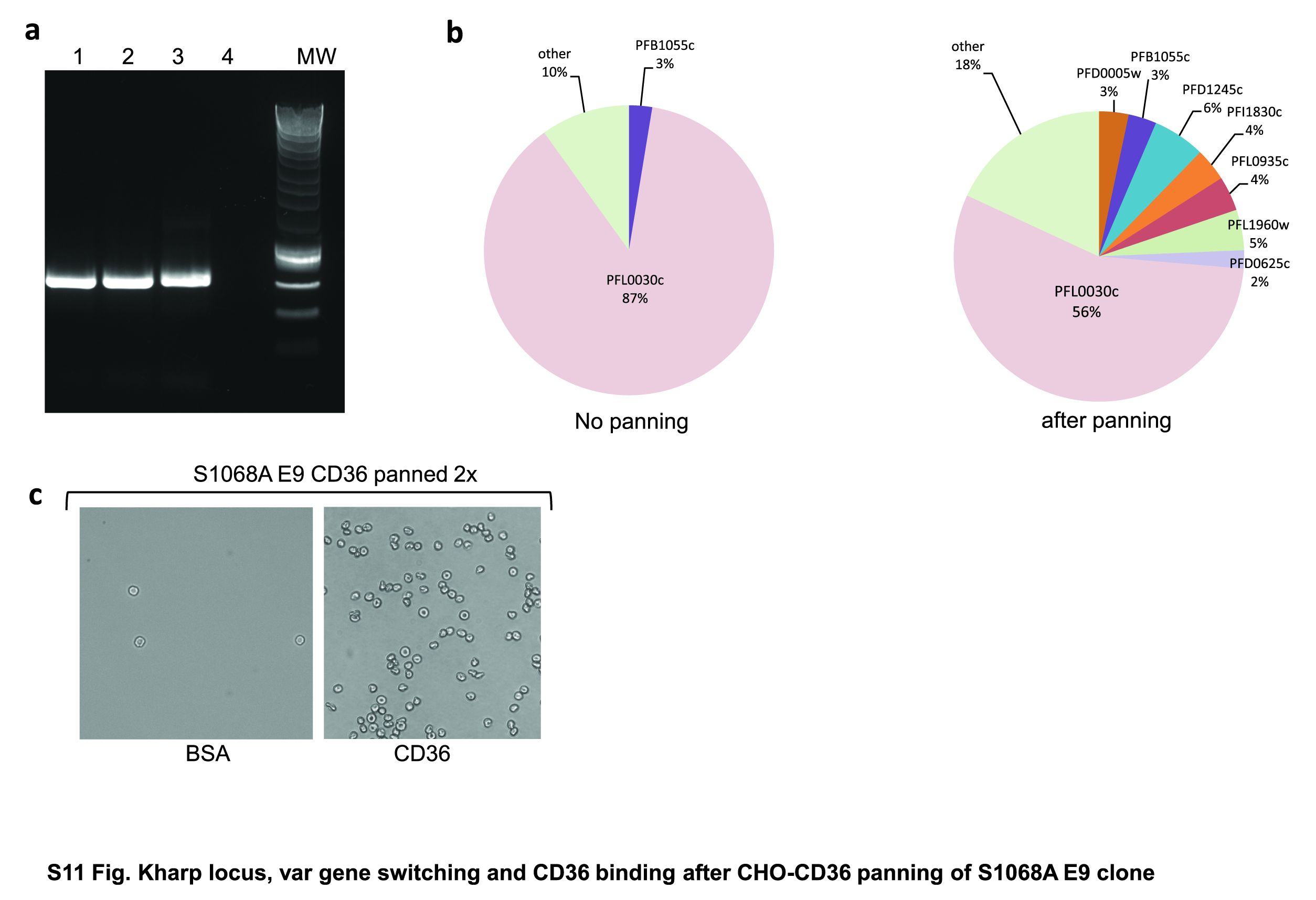

Supplement: S11 Fig — (a) Kharp locus PCR amplification. PCR was performed using GXL polymerase and kharp specific primers on genomic DNA (60ng) of NF54CSA WT parental line and NF54 S1068A mutant clones E9 and A10. Lane1: Clone VAR2CSA S1068A A10; lane2: Clone VAR2CSA S1068A E9; lane 3: WT parental line; lane 4: H2O negative control; Lane 5: Molecular Weight. (b) Var transcriptional profile of S1068A clone E9 before and after 2 rounds of panning on CHO cells expressing CD36 shown by qPCR. Transcriptional levels of each var genes were normalized with the housekeeping gene, seryl-tRNAtransferase. (c) CD36 cytoadhesion assay of S1068A clone E9 IEs after 2 rounds of panning on CHO cells expressing CD36. (TIF) [file ppat.1012861.s011.tif]
