# Supplementary material for: Casein Kinases 2-dependent phosphorylation of the placental ligand VAR2CSA regulates Plasmodium falciparum-infected erythrocytes cytoadhesion
Source: PLoS Pathog. 2025 Jan 13;21(1):e1012861. doi: 10.1371/journal.ppat.1012861 (PMC11761665; doi:10.1371/journal.ppat.1012861)
Supplement: Attachment — Submitted filename: Answers to reviewers.docx [file ppat.1012861.s013.docx]

Reviewer's Responses to Questions

**Part I – Summary**

Please use this section to discuss strengths/weaknesses of study, novelty/significance, general execution and scholarship.

**Reviewer #1:** The submission from Dorin-Semblat and colleagues describes a series of experiments designed to identify the kinase or kinases responsible for phosphorylating an important virulence factor in the malaria parasite Plasmodium falciparum, a protein called VAR2CSA. The authors also provide evidence for a role of phosphorylation in protein trafficking and transport to the infected RBC surface. VAR2CSA is recognized at the primary protein responsible for pregnancy associated malaria and is a high-profile candidate as a potential vaccine that could protect women from this important complication of infection by P. falciparum. Understanding the role of phosphorylation of the exposed portion of the protein therefore has important implications for both our basic understanding of protein processing and trafficking as well as for possible vaccine development.

The authors have built on previous studies from their group and others that detected phosphorylation of VAR2CSA. Here they provide additional evidence that both human and parasite CK2 can phosphorylate VAR2CSA and they identify the specific residues that serve as substrate for the kinases. They also generate a mutant parasite line in which a specific residue has been altered and show that phosphorylation of this residue is important for translocation of VAR2CSA to the infected cell surface. Overall, the manuscript is well written, and the experiments are well designed. There are however a few instances in which additional controls would make the conclusions more convincing.

**Reviewer #2:** In this manuscript, Dorin-Semblat et al. report that Casein Kinase 2a (CK-2a) is involved in the phosphorylation of Variant Surface Antigen Var2CSA. Var2CSA is exported by the parasite to the surface of host erythrocyte surface and is involved in P. falciparum sequestration and cytoadherence with chonodritin sulfate A (CSA). The authors demonstrate that both human and parasite Casein Kinase 2a phosphorylate recombinant or immunoprecipitated Var2CSA. The phosphorylation sites for CK2a on Var2CSA were also identified. They also demonstrate that CK2a inhibitors block adhesion to CSA and erythrocyte. These inhibitors also impair the phosphorylation of Var2CSA by the parasite and erythrocyte lysate. Finally, they generate a S>A mutant of one of the phosphorylation sites of VAR2CSA in the parasites and demonstrate that the mutant (S1068A) parasites exhibit reduced binding to CSA.
In my opinion the work, largely lacks novelty as previous studies have already demonstrate that CK2 inhibitors prevent VAR2CSA phosphorylation (Hora et al., JBC 2009). Further there is no direct genetic evidence that PfCK2a indeed phosphorylates Var2CSA in the parasite, which is needed to demonstrate the link between the two as kinase inhibitors can be non-specific. However, experiments performed with S1068A mutant parasites are interesting which seem to suggest that phosphorylation of this site may be important for erythrocyte adherence. Unfortunately, this part of the manuscript is not well-developed.

We would like to thank the reviewers for their expert critiques and thoughtful suggestions. We believe that the present version of the manuscript has addressed all the concerns raised by both reviewers and that the revisions have improved the quality and clarity of our manuscript.

We believe that we answered all the reviewer’s comments and performed the additional controls requested. We also provide further evidences that the phosphorylation of the S1068 residue is important for trafficking VAR2CSA on the surface of the infected erythrocytes.

**Part II – Major Issues: Key Experiments Required for Acceptance**

**Reviewer #1:**

1. In the section entitled “Endogenous PfCK2α phosphorylates rVAR2CSA”, the authors use antibodies against PfCK2α to immunoprecipitate the protein from infected RBCs, then show that it can phosphorylate recombinant VAR2CSA. There is a formal possibility that these antibodies could also recognize human CK2 or that the human enzyme could otherwise contaminate the precipitated protein, and thus the phosphorylation of recombinant VAR2CSA in the assay could be due to the human protein rather than the parasite protein. While this is unlikely, it could be ruled out by including an additional control of immunoprecipitation from uninfected RBCs.

This is a good point. The rabbit anti-PfCK2 antibody has been raised against a peptide that is not present in the human protein sequence, precisely to prevent cross-reactivity (Supplementary figure 3). Nonetheless we have performed a new immunoprecipitation with uninfected red bloods cells lysates using antibodies directed against the Human and Plasmodium enzymes and we analyzed the immunoprecipitated material by western blots with both antibodies. We included additional controls in the western blot such as recombinant kinases GST-PfCK2a (~65kDa) and MBP-Hu CK2a (~78kDa).

As shown in the Western blots of Supplementary figure 3:

1) Hu CK2a is not immunoprecipitated from uRBC lysates by the anti-PfCK2a antibody. Furthermore, anti PfCK2a recognizes the recombinant Plasmodium kinase but not the recombinant HuCK2a indicating that the anti Pf kinase antibodies do not cross react with the RBC kinase (again, they were designed to prevent such cross-reactivity).
2) Human CK2 is immunoprecipitated by an anti Hu CK2a lysates as expected. This antibody does recognize the recombinant human kinase but not the recombinant GST-Pf CK2a.

Overall, these experiments exclude cross-reactivity and resolve the issue raised by the reviewer.

The following paragraph was inserted into the revised manuscript: “To verify that the anti-PfCK2α antibody does not cross-react with the Human CK2α, we performed an immunoprecipitation with uninfected RBCs lysates. While the anti-Human CK2α immunoprecipitated the RBC kinase, no immunoprecipitation of the Human kinase was observed with the anti-PfCK2α. Furthermore, no cross-reactivity was observed by western blot on recombinant GST-PfCK2α (~65kDa) and MBP-HuCK2α (~78kDa). Taken together these results confirmed the specificity of both antibodies (Supplementary figure 3).”

2. In the section entitled “Effect of S1068A mutation on rVAR2CSA DBL1-6 in vitro binding” the authors perform binding assays with wildtype and mutant VAR2CSA. The authors’ model is that this amino acid is important for phosphorylation, which can affect trafficking, binding or both. Here they investigate a role for binding, but it appears they did not include a kinase to phosphorylate this site. The authors show a slight decrease in binding when comparing unphosphorylated S1068 to A1068, but isn’t the relevant comparison between phosphorylated S1069 to A1068?

We agree with the reviewer. It is indeed likely that the S1068 residue of the VAR2CSA protein produced in HEK cells is not fully phosphorylated.

Since we have shown by Mass spectrometry that the Human CK2a phosphorylates the S1068 residue, we decided to assess the binding of VAR2CSA to CSA at 4 different concentrations after addition or not of human CK2 in the presence of ATP.

Interestingly, we observed in 3 independent experiments an increased binding of VAR2CSA to CSA when VAR2CSA was preincubated with the kinase plus ATP indicating that CK2 phosphorylation enhances VAR2CSA binding to CSA, supporting the difference observed between the S1068 and A1068. (Figure 6b)

The following paragraph was inserted into the revised manuscript: “Since we have shown by mass spectrometry that the HuCK2α phosphorylates the S1068 residue and that the phosphorylation could contribute to VAR2CSA binding to CSA, we assessed the binding of rDBL1-6 to CSA at 4 different concentrations after addition or not of human CK2a in the presence of ATP. Interestingly, we observed in 3 independent experiments a significant increase in the binding to CSA (p= 0.018; 0.03; 0.0008 and 0.0002 for respectively 0.2, 0.5, 1 and 2µg/ml rDBL1-6) when VAR2CSA was preincubated with the kinase plus ATP compare to the condition without kinase, indicating that HuCK2α mediated VAR2CSA phosphorylation enhances binding to CSA (Figure 6b), supporting the difference observed between the S1068 and A1068 recombinant proteins.”

3. In the last section of the Results, the authors generate a mutant parasite line in which they have inserted the S1068A mutation and demonstrate that VAR2CSA is not trafficked to the RBC surface. This is a particularly interesting result and adds significantly to the paper. However, it is common for cultured parasites to lose surface expression of PfEMP1, something that is frequently observed in lines of 3D7, for example. It is also worth noting that inhibition of CK2 activity with TBCA or DMAT did not affect surface expression of VAR2CSA. Therefore, validating this phenotype is important. The standard method for validation of a mutant line is via complementation: reverting the mutation and rescuing the wildtype phenotype, in this case VAR2CSA surface expression. An alternative would be to isolate additional clones from an independent transfection and showing the identical phenotype.

This is again a very good point raised by the reviewer: cultured parasites can indeed lose surface expression of PfEMP1 and notably knobs on the IEs surface. To monitor this, we regularly select knob-positive parasites using plasmion. To answer that comment, we verified the presence of KHARP by PCR and found no deletion in two clones. In addition, to ensure that the mutant clone E9 has not lost the ability to translocate other PfEMP1s than VAR2CSA at the surface, we performed 2 rounds of panning of the mutant cell line on CHO cells expressing CD36, another PfEMP1s receptor. We showed that after2 rounds of panning, VAR2CSA transcripts decrease from 90 % to 57%. Other groups of VAR transcripts were expressed suggesting that other PfEMP1s variants expressed at the surface of the S1068A IEs can therefore bind to CD36 receptor present on the CHO membrane and also on purified CD36 coated on plastic (Supplementary Figure 11).

As requested, we also assessed the mutant phenotype of another clone (A10). Using several approaches: qPCR, cytometry, cytoadhesion, this mutant reveals the same phenotype than the E9 clone described in the paper: No VAR2CSA on the IEs surface (Supplementary figure 10)

The following paragraph was inserted into the revised manuscript:

“We assessed the phenotype of another S1068A clone (A10). Using several approaches (qPCR, cytometry, IFA and IEs cytoadhesion) this mutant reveals the same phenotype as the E9 clone with the lack of VAR2CSA on the IEs surface **(Supplementary figure 10)**. To ensure that the S1068A transgenic parasites PfEMP1 export machinery to the IEs surface was still fully functional, we verified the presence of the *kharp* locus associated to knobs formation in both E9 and A10 clones and performed 2 rounds of S1068A IEs E9 clone panning on CD36-expressing CHO745 cells **(Supplementary Figure 11)**. No *kharp* locus deletion was observed in both mutant cell lines. Furthermore, parasites selected for CD36 adhesion, displayed a decreased expression of *var2csa* (90% to 57%) while expression of multiple group B and C var genes was observed, indicating that var gene switching is not impaired and that CD36-binding PfEMP1 can be exported to the IEs surface. IEs cytoadhesion to CD36 receptor was also verified by static binding as shown in Supplementary Figure 11. Taken together these results indicate that the S1068A E9 clone has an intact PfEMP1 export machinery.”

**Reviewer #2:**

1. Do CK2 inhibitors block parasite growth?

Little is known about the inhibition of parasite growth by both compounds. In the study of Hitz E et al (Communications biology 2023), four commercially available cell-permeable ATP-competitive inhibitors of human CK2α (Quinalizarin, TTP 22, TBB and DMAT) were tested for their ability to reduce or block parasite multiplication in a [3H]-hypoxanthine incorporation assay. None of these compounds displayed potent activity. Indeed, in their cell-based assay, TBB showed poor activity (IC50 > 50 µM) and for DMAT they determined an IC50 of 15.8µM Consistent with this, we did not observe any difference in parasite maturation from rings to trophozoites stages in the presence of two of these inhibitors (DMAT and TBCA) at 50µm (data not shown).

2. Figures 2-4: Results in these figures demonstrate that human/Pf/CK2 interact with Var2CSA and phosphorylate it in vitro. Several of the experiments demonstrate the same result therefore are repetitive.

Indeed, we performed multiple assays to demonstrate that both the Human and parasite CK2 phosphorylate VAR2CSA. We would argue that reinforcing data using several methods consolidate the conclusion that can be drawn from these experiments. We would be happy to move some of the experimental results to Supplementary Information if needed.

The experiments performed with total cell lysates as kinase source are "crude" as several other kinases can phosphorylate VAR2A and the inhibitors can block them especially at high concentrations. Therefore, these may be excluded especially when kinase assays have been performed with purified recombinant hu/PfCk2 or with IP, which provide direct evidence of phosphorylation by the kinase although it is only in vitro.

Having these “crude” experiments as a first step towards the demonstration that both human and *P. falciparum* CK2 phophorylate VAR2CSA should in our view be shown in the manuscript.

We agree that the inhibitors could block directly or indirectly other kinases present in the crude extract. This was mentioned at the end of the Results section (page 6): “These results suggest that CK2 from the red blood cell and/or *P. falciparum* are candidate kinases implicated in the phosphorylation of VAR2CSA”. Furthermore, in the discussion (page 14), we wrote: “we cannot exclude that this effect (inhibition of VAR2CSA phosphorylation) by both compounds may be mediated indirectly (for example, by kinases regulated by CK2α)”.

3. The concentration of TBCA/DMAT used in experiments is in 10-50uM, which seems to be too high. What is the IC50 of these inhibitors against human and PfCK2.

In Hora et al, JBC 2009, the authors used various CK2 inhibitors at 100µM, including TBCA/DMAT, to assess *P falciparum* adhesion. Engelberg K et al, Biochemistry J 2013, used TBCA to inhibit Rh2b phosphorylation in a dose-dependent manner. In presence of parasite lysate, 10 μM of TBCA reduced Rh2b phosphorylation whereas 50 μM entirely eliminated phosphorylation. Based on these two manuscripts, we decided that the optimal concentration to be used in this study should be in that range (10-50 μM).

With respect to the IC50 of CK2 inhibitors: in our Supplementary Figure 6, we show a significant inhibition of VAR2CSA DBL1-6 phosphorylation by recombinant PfCK2a with 2µM TBCA. Another CK2 inhibitor (TBB, a structural analogue of DMAT), displays an IC50 around 2.5 µM against purified PfCK2 activity using a peptide substrate (Holland et al, Eukaryotic Cell 2009);

More studies on the inhibitory effect of this class of compounds have been performed with human CK2a activity. Pagano M A et al (Chem Biochem 2007) monitored the endogenous activity of CK2a in Jurkat cells after 24 hours of treatment with increasing concentrations of TBCA and showed a 50% reduction around 10µM of TBCA. In another study ([Biochemical and Biophysical Research Communications](https://www.sciencedirect.com/journal/biochemical-and-biophysical-research-communications) 2004) human CK2a activity was drastically reduced upon treatment with 5 and 10µM of DMAT. In our study (Supplementary Figure 6) VAR2CSA phosphorylation by human CK2a is drastically inhibited by 2µM DMAT

As expected, the IC50 of both inhibitors against the recombinant or purified human enzyme was around 0.15µM, much lower than that in cell-based assays (and also lower than the IC50 against purified PfCK2, see above).

4. Do Var2CSA and CK2- co-immunoprecipitate and co-localize in the parasite and/or IE?

We performed a VAR2CSA immunoprecipitation experiment on purified IEs membranes, followed by Western Blot using anti-PfCK2a and anti-human CK2a. Although VAR2CSA was immunoprecipitated, we did not observe co-immunopurification of PfCK2a or Hu CK2a. This result is shown later in **Part III.** (see answer to point # 1 of Reviewer1 (minor issues))

5. Given that the ectodomain of Var2CA is phosphorylated by CK2, how does it come in contact with PfCK2? Does it get phosphorylated in the parasite before it is trafficked to the host?

This is indeed a good question. PfCK2α is expressed at all erythrocytic stages, with greater expression in mature stages, where it is present in the nucleus and cytoplasm (Dastidar et al BMC Biology 2023; Hitz et al Communications biology 2021). Furthermore, four PfCK2a peptides were found in a proteomic study aiming to identify IRBC surface proteins, using surface biotinylation (Florens et al Molecular and Biochemical Chemistry 2004). Therefore, PfCK2 might be exported. Phosphorylation of VAR2CSA can possibly occur during trafficking of the proteins to the RBC surface (as they are predicted to share the same trafficking compartments); it cannot be excluded that CK2 is a true exokinase and may phosphorylate VAR2CSA in the extracellular medium

6. Figures 5-7: S1068 present in the CIDR is identified as the target phosphorylation site for hu/PfCK2a. There seems to be only a modest decrease in binding of S1068A mutant line to CSA. Statistical analysis of the data provided in Figure 6 should be performed.

Statistical analysis was performed and is now provided as requested using the method described in the following web link: <https://www.graphpad.com/support/faqid/2031/>. As shown, the statistical analysis of the data displays a p value around 0.0001

7. Figure 7: The finding that trafficking of the S1068A mutant to the host RBC is impaired is interesting. However, more experiments need to establish that S1068A phosphorylation is critical for the export of Var2CSA to the host, which in turn is important for adhesion.
Here are a few suggestions:

a. The quality of IFA provided in Fig. 7c should be better and an additional marker for PVM and RBC surface may be included.

New IFAs with an improved quality have been performed to support this finding. Furthermore, as requested, a marker of the erythrocyte membrane, Glycophorin A, has been included in the analysis. In live-IFA, surface labelling of the red cell membrane showed that the typical punctuated pattern of VAR2CSA clearly colocalizes with GPA in the wild-type, whereas no VAR2CSA labeling was detected on the surface of the mutant cell line. The same results have been obtained with a second, independent mutant clone (Supplementary Figure 10). Labelling permeabilized cells with an anti-Skeleton Binding Protein 1(SBP1) antibody to visualize the Maurer’s Clefts, an intermediate trafficking compartment for PfEMP1 translocation to the host cell membrane showed that in both cell lines, VAR2CSA and SBP1 display the same staining pattern, suggesting that VAR2CSA is present in this intermediate organelle but the final stages of its export to the IEs surface is impaired in the mutant cell line.

b. Figure 7D Western blot shows that S1068A is expressed at higher levels that WT Var2CSA. Was IE or only parasite lysate was used for this experiment? It may be good to fractionate RBC and parasite fractions and compare the WT and S108A mutant in these fractions.

In Figure 7 D, there is no significant difference in the fold changes between VAR2CSA and S1068. We performed a quantification of the intensity of the bands of VAR2CSA and anti PfNapL western blots using the Image J software (this is now shown in Supplementary Figure 8. A 10X fold change was found between the intensities of VAR2CSAS1068A and VAR2CSA WT bands while an 8.5-fold change was observed between VAR2CSA WT and S1068A bands of the internal control (PfNapL). In this experiment we used total parasites lysates including soluble and membrane fractions. Fractionation of the different compartments is difficult to perform and time consuming. Therefore, we couldn’t assess it in this manuscript

c. BrefeldinA, which blocks protein transport, can be used to compare the trafficking of mutant and WT Var2CSA. In addition, co-localization with CK2 can be performed.

Indeed, this is another experiment that could be performed. We did not implement it in view of the additional GPA/SBP1 co-localization data (see above). Furthermore, our PfCK2 antibody is unfortunately not adequate to perform IFA.

**Part III – Minor Issues: Editorial and Data Presentation Modifications**
Please use this section for editorial suggestions as well as relatively minor modifications of existing data that would enhance clarity.

**Reviewer #1:**

1. Similar to point one above, in the section entitled “Native VAR2CSA is phosphorylated by rHuCK2α”, the authors show that immunoprecipitated VAR2CSA can be phosphorylated by recombinant human CK2. However, they do not include a control to show that the immunoprecipitated protein is not contaminated with parasite CK2 (or any other kinase activity). This is particularly relevant considering that later in the manuscript they show that both the human and parasite kinases can be co-immunoprecipitated by anti-VAR2CSA antibodies. A simple control would be to perform the radiolabeling reaction with immunoprecipitated VAR2CSA but in the absence of recombinant CK2. The authors performed an equivalent control in the following section when they incubated immunoprecipitated VAR2CSA with an enzymatically dead recombinant PfCK2α and observed no phosphorylation, thus demonstrating that their immunoprecipitated protein does not contain significant contaminating kinase activity. It would be good to include a similar control in this experiment or at least mention the subsequent result.

Regarding the above comment (highlighted in yellow), we have not shown in this manuscript that the human and parasite CK2a co-immunoprecipitate using VAR2CSA antibodies. The interaction was shown in this study by a pulldown approach with purified recombinant enzymes (His VAR2CSA DBL1-6 and MBPHu CK2a /GSTPfCK2a). However, in response to the point # 4 made by Reviewer 2, we performed supplementary immunoprecipitation experiments described below to confirm that we did not observe a co-immunoprecipitation of endogenous VAR2CSA with the native kinases.

Figure Legend: **PfCK2a or Hu CK2a do not co-immunoprecipitate with VAR2CSA.**

VAR2CSA was immunoprecipitated from lysates of NF54VAR2CSA IEs using a rabbit polyclonal antiPfVAR2CSA antibody. The immunoprecipitated material was analyzed by western blots with anti VAR2CSA antibody and with the same CK2a antibodies. **(A)** Anti VAR2CSA (top panel) and anti Hu CK2a (lower panel) western blots. Lane1: MW; lane2: input membrane fraction NF54VAR2CSA IEs; lane3: VAR2CSA immunoprecipitation; lane 4: r VAR2CSA DBL1-6 (100ng) and rGSTPfCK2a (100ng). **(B**) Anti VAR2CSA (top panel) and anti Hu CK2a (lower panel) western blots. Lane 1: input soluble fraction NF54VAR2CSA IEs; lane2: input membrane fraction NF54VAR2CSA IEs; lane3: VAR2CSA Immunoprecipitation from soluble fraction; lane 4: VAR2CSA Immunoprecipitation from membrane fraction; lane 5: rVAR2CSA (50ng); lane 6: rHuCK2a (50 ng); lane7: MW. Left panels display the stain free gels and right panels correspond to the western blots.

As requested by the reviewer, an immunoprecipitation control of VAR2CSA in the absence of recombinant CK2a has been actually performed. A phosphorylation assay was carried (without exogenous kinase) after VAR2CSA immunoprecipitation from NF54VAR2CSA iRBC. This is now shown in Supplementary Figure 4. The autoradiogram shows no signal in the control without kinase (lane 6) compared to the signal obtained after radiolabeling of immunoprecipitated VAR2CSA with the recombinant HuCK2a (lane 3).

2. Minor corrections: throughout the manuscript, the word “var” is sometimes italicized and sometimes not. In Figure 7, panel B, the word “VAR2CSA” is misspelled next to S1068A.

This has been corrected

Reviewer #2: The quality of images and statistical analysis needs to be done as indicated in my comments

Quality of images has been improved and statistical analysis has been done as requested
